# Scanning Spin Probe Based on Magnonic Vortex Quantum Cavities

**DOI:** 10.1021/acsnano.3c06704

**Published:** 2024-01-25

**Authors:** Carlos A. González-Gutiérrez, David García-Pons, David Zueco, María José Martínez-Pérez

**Affiliations:** † Instituto de Nanociencia y Materiales de Aragón (INMA), CSIC-Universidad de Zaragoza, Zaragoza ES-50009, Spain; ‡ Department of Physics and Applied Physics, University of Massachusetts, Lowell, Massachusetts 01854, United States; ¶ Instituto de Ciencias Físicas, Universidad Nacional Autónoma de México, Av. Universidad s/n, Cuernavaca, Morelos 62210, México

**Keywords:** quantum sensing, electron paramagnetic resonance imaging, magnetic vortex, quantum magnonics, magnetic sensor

## Abstract

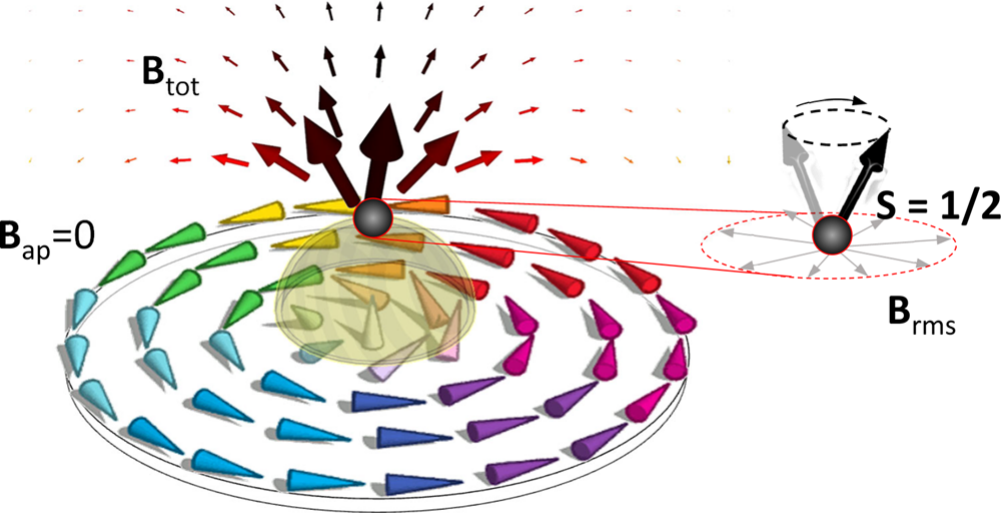

Performing nanoscale scanning electron paramagnetic resonance (EPR) requires three essential ingredients: First, a static magnetic field together with field gradients to Zeeman split the electronic energy levels with spatial resolution; second, a radio frequency (rf) magnetic field capable of inducing spin transitions; finally, a sensitive detection method to quantify the energy absorbed by spins. This is usually achieved by combining externally applied magnetic fields with inductive coils or cavities, fluorescent defects, or scanning probes. Here, we theoretically propose the realization of an EPR scanning sensor merging all three characteristics into a single device: the vortex core stabilized in ferromagnetic thin-film discs. On one hand, the vortex ground state generates a significant static magnetic field and field gradients. On the other hand, the precessional motion of the vortex core around its equilibrium position produces a circularly polarized oscillating magnetic field, which is enough to produce spin transitions. Finally, the spin–magnon coupling broadens the vortex gyrotropic frequency, suggesting a direct measure of the presence of unpaired electrons. Moreover, the vortex core can be displaced by simply using external magnetic fields of a few mT, enabling EPR scanning microscopy with large spatial resolution. Our numerical simulations show that, by using low damping magnets, it is theoretically possible to detect single spins located on the disc’s surface. Vortex nanocavities could also attain strong coupling to individual spin molecular qubits with potential applications to mediate qubit–qubit interactions or to implement qubit readout protocols.

## Introduction

Electron paramagnetic resonance (EPR) is widely used in chemistry, physics, medicine, and material science to characterize the electronic structure of magnetic molecules and impurities.^1^ This has important applications in the study of organic and inorganic free radicals, colored centers in crystals, tissue oxygenation, and archeological dating, to give a few examples. Similarly to the physics of nuclear magnetic resonance imaging,^2^ EPR can be combined with nanoscopic field gradients to detect spatially distributed spins.^3^

The technological interest in EPR has led to the development of sophisticated and sensitive detection methods. These include optical techniques, such as using optically active atomic defects,^4−7^ electrical detection of magnetic signals using scanning tunneling microscopy probes,^8−10^ or mechanical sensing based on magnetic resonance force microscopy.^11,12^ Among the inductive methods, pickup coils and superconducting quantum interference devices (SQUIDs) have been employed to characterize resonant phenomena in small paramagnetic crystals.^13,14^ However, the most widespread inductive-EPR readout technique uses transmission lines and cavities.^15−17^ This latter approach offers the advantage of confining light in the space domain, yielding increased light–matter interactions. In addition, the cavity’s quality factor can be further enhanced when using superconducting coplanar resonators instead of metallic three-dimensional cavities,^18^ yielding increased visibility. This idea is exploited in circuit Quantum Electrodynamics (QED),^19^ allowing, e.g., the manipulation and interrogation of superconducting or magnetic qubits^20^ and quantum sensing of small amounts of spins.^21−29^

The light–matter coupling factor (g) depends on the amplitude of the (position-dependent) root-mean-square (rms) vacuum magnetic field fluctuations in cavity **B**_rms_(**r**). The latter increases for decreasing mode volume, favoring the use of small cavities. However, downsizing is limited by the maximum operating frequency (1–20 GHz, typically) and the impedance of the resonator. Large coupling factors can be reached by fabricating nanoscopic constrictions at the central transmission line in superconducting coplanar resonators.^30−32^ This approach keeps the total length of the cavity while reducing the other two dimensions, yielding strong focusing of the rms vacuum field fluctuations in nanometer regions around the central conductor. By doing so, large coupling factors of *g*/2π ∼ 1 kHz per spin have been demonstrated.^33^ Alternatively, low impedance LC-resonators allow increasing the amplitude of the magnetic compared to the electric field fluctuations, also yielding increased couplings.^22,23,26,29,34^

In addition to photons, the solid state offers a wide variety of bosonic excitations that can be emitted or absorbed such as, e.g., quantized spin waves or magnons.^35−38^ In what follows, we will use *g* to denote both the spin–photon and spin–magnon coupling. Magnonic cavities could be used to perform spin qubit readout or to mediate spin–spin interactions,^39−49^ offering the advantage of increasing the coupling by operating at reduced wavelengths (compared to electromagnetic resonators of the same frequency). This is possible since spin wave modulation is limited only by the lattice constant of the ferromagnet, allowing the downsizing to the nanometer range. This significantly decreases the mode volume and results in substantial spin–magnon couplings. For example, quasi-homogeneous spin waves in saturated ferromagnets have been proposed to substitute superconducting cavities.^50,51^ Using nanoscopic yttrium–iron–garnet (YIG) spheres, such an approach shall provide strong coupling to individual free electrons, even reaching *g*/2π ∼ 1 MHz. Interestingly, whispering gallery modes in relatively large vanadium tetracyanoethylene (V[TCNE]_*x*_) discs would yield a sizable spin–magnon coupling to individual NV centers.^52^ This is so for the relevant mode volume is given by the disc’s thickness *t* and the angular index magnon mode. In this way, a very encouraging *g*/2π ∼ 10 kHz can be obtained with a *R* = 500 nm, *t* = 100 nm out-of-plane saturated disc at 1.3 GHz. However, both approaches do require an externally applied bias field *B*_ap_ with a double purpose: to saturate the ferromagnetic volume and to tune its resonance frequency with that of the spins. Interestingly, magnons can be confined in peculiar flux-closure configurations at *B*_ap_ = 0. This is the case of magnetic vortices that are easily stabilized in thin-film ferromagnetic structures with lateral size between a few 10 nm up to several micrometers.^53,54^ Minimization of the magnetostatic energy yields a circular in-plane arrangement of spins with a nanoscopic out-of-plane magnetization core in the center. The vortex core modifies the spin-wave spectrum of the ferromagnet, yielding additional resonant modes in the absence of externally applied fields.^55,56^

Here, we compare the spin coupling to microwave resonators with that resulting from magnonic cavities (both saturated ferromagnets and flux-closure states). In the case of magnons, we base our calculations on three archetypical materials, common in the field of quantum magnonics:^57−59^ On the one hand, a ferrimagnetic garnet with record low damping but low saturation magnetization (YIG, with Gilbert damping parameter α ∼ 10^–4^). On the other hand, two ferromagnets with larger magnetization but higher damping, i.e., Co_25_Fe_75_ alloy (CoFe, with α ∼ 10^–3^) and Permalloy (Py, with α ∼ 0.5 × 10^–2^), the most widely used soft-ferromagnet for spin-wave-based applications. We first demonstrate that the coupling to magnons is more than 2 orders of magnitude larger than that resulting from cavity photons. Although being of the same order, using vortices has important advantages over saturated ferromagnets such as the absence of a biasing magnetic field, independence from the saturation magnetization (*M*_sat_), and the capability to manipulate the vortex’s position. This ability enables scanning over a range of tens of nanometers. In summary, our findings highlight the potential of flux-closure states compared with other systems, demonstrating that the vortex core can be operated as a nanoscopic scanning EPR probe. Coupling the ferromagnetic disc to a superconducting circuit, our approach could be used to spatially resolve the location of single spins distributed over the surface of the disc.

## Results

EPR is a resonant phenomenon, and regardless of the use of superconducting or ferromagnetic cavities, it must satisfy two conditions. The first criterion is energetic: the frequency ω_0_ of the rf field produced by the cavity needs to match the energy difference between the Zeeman-split spin levels. In the case of free *S* = 1/2 spins, the latter means ω_*s*_ = γ_*e*_*B*_tot_(r) = ω_0_. Here, γ_*e*_/2π = 28 GHz/T and *B*_tot_(r) ≡ |**B**_tot_(r)| is the total (position-dependent) magnetic field that contributes to the Zeeman splitting. This criterion leads us to the definition of the *resonance window*, i.e., the region in space where spins are resonant with the cavity (see Methods section). We highlight that the resonance window will depend only on the modulus of the (position-dependent) **B**_tot_(r).

The second condition is imposed by the geometry of the experiment: spin transitions can be induced only by the components of the vacuum field fluctuations that are perpendicular to the quantization axis of the spin. In the case of free *S* = 1/2 spins, the latter is parallel to **B**_tot_. This determines the strength of the spin-photon coupling *g*. To quantify the coupling, it is convenient to introduce the set of mutually orthogonal vectors **B**_rms,*j*_, *j* = 1, 2, 3 so that **B**_rms,3_∥**B**_tot_. Thus, the other two components can induce spin transitions, allowing us to define . Consequently, in the case of *S* = 1/2, the spin-photon coupling is given by *g* = μ_B_*B*_rms_ with μ_B_ being the Bohr magneton. [Note: This is a direct consequence of the choice of the orthogonal vectors **B**_rms,*j*_ and the formula *g* = *g*_*e*_μ_B_⟨0|**S**·**B**_rms_(r)|1⟩. Here, *g*_*e*_ is the gyromagnetic factor and {|0⟩, |1⟩} are the two states-induced spin transitions.] We highlight that *g* will depend both on the intensity of the vacuum field fluctuations and also on the position-dependent distribution of its components with respect to **B**_tot_(r).

### Coupling to Spins, Comparison between Photons and Magnons

**Figure 1:**
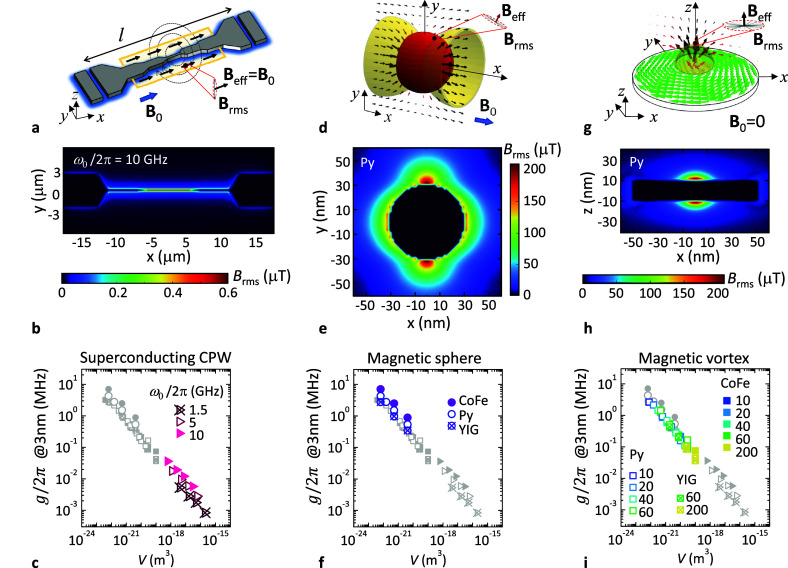
Comparison between electromagnetic cavities (coplanar waveguide) and magnonic resonators (saturated sphere and magnetic vortex). (a, d, and g) Scheme of the resonators. **B**_ap_ is the external bias field (big blue arrow). **B**_tot_ = **B**_ap_ + **B**_stray_ is the total field (black arrows), with **B**_stray_ the demagnetizing field and **B**_rms_ the zero point fluctuation field (gray arrows inside the red dashed circle). The yellow regions are the resonance windows. Below we plot the spatial distribution of *B*_rms_ created by zero point current fluctuations in the electromagnetic cavity (b) and by the zero point magnetization fluctuations in a Py sphere (e) and a Py disc (h) of similar volume. Bottom panels (c, f, and i) are the calculated *B*_rms_ at 3 nm above the resonators at the position of the white dot in panels (b), (e), and (h). Simulations evidence the expected dependence *B*_rms_ ∝ *V*^1/2^.

We start by analyzing the spin–photon coupling in electromagnetic cavities. This will help us compare it with the case of magnons, which will be discussed below. We focus on the particular case of a superconducting coplanar waveguide formed by interrupting an open transmission line by two gap capacitors as sketched in Figure 1a. The external magnetic field **B**_ap_ = **B**_tot_ shall be ideally applied along the transmission line axis so that, in this case, *B*_rms_ = |**B**_rms_|. Under these circumstances, all spins can satisfy the resonance condition and are susceptible to be detected. In this case, the volume *V* of the resonator is the total length *l* (see Figure 1a) multiplied by the cross-section of the central transmission line. *l* is fixed by the operating frequency ω_0_ which, together with the circuit’s impedance *Z*_0_, sets the intensity of the zero-point current fluctuations^60^. Using electromagnetic waves imposes a lower limit on the total length of the cavity, *l* > λ/2 = 2*πv*/ω_0_, where λ and *v* are the wavelength and phase velocity, respectively. Working at high frequencies allows increasing the coupling by reducing the volume, but this is limited to ω_0_/2π < 10–15 GHz due to technical reasons. Decreasing *V* can only be accomplished by decreasing the cross-section which has the effect of focusing the vacuum field fluctuations while keeping *i*_rms_ unchanged. Figure 1b shows the spatial distribution of *B*_rms_ for a ω_0_/2π = 10 GHz resonator of thickness 50 nm and different widths (5 μm, 500 nm, and 50 nm). Figure 1c shows *B*_rms_ vs *V* calculated at 3 nm above the central conductor for different values of ω_0_ and the cross-section (10 × 10 nm^2^, 20 × 20 nm^2^, 35 × 35 nm^2^, and 70 × 70 nm^2^). From our simulations we see that the spin-photon coupling between free spins and *Z*_0_ = 50Ω-resonators is limited to the range 1–50 kHz, in agreement with recent experiments.^33^

Alternatively, spin waves propagate at much lower velocities, allowing λ to decrease while working at frequencies in the 1–20 GHz range. This is the basic idea behind the use of magnonic cavities. One basic example to start with is the quasi-homogeneous Kittel mode (*k* = 2π/λ = 0) excited in isotropic ferromagnets, e.g., spheres. Zero point magnetization fluctuations in the ferromagnet produce zero point field fluctuations on the outside. Figure 1e shows the spatial distribution of *B*_rms_ for a saturated Py sphere for which impressive values of 200 μT can be reached. Figure 1f shows *B*_rms_ at 3 nm above the surface of spheres of different sizes (*R* = 25 nm, 50 nm, and 100 nm) and materials (CoFe, Py, and YIG). The intensity of field fluctuations does not depend on *B*_ap_ and satisfies *B*_rms_ ∝ *V*^–1/2^*M*_sat_^1/2^, with *V* the volume of the ferromagnet. As it can be seen, the resulting couplings *g* = μ_B_*B*_rms_ will be more than 2 orders of magnitude larger than those achievable with superconducting resonators, even approaching the 10 MHz range.

As a drawback, this approach requires unavoidably the use of an external bias field **B**_ap_ that serves to saturate the ferromagnet and to tune its resonance frequency ω_K_ = γ_*e*_*B*_ap_. In addition, unlike the case of a superconducting resonator, paramagnetic spins located close to the sphere’s surface will feel a strongly nonhomogeneous magnetic field **B**_tot_ = **B**_stray_ + **B**_ap_ with **B**_stray_ the demagnetizing field created by the sphere itself (see arrows in Figure 1d). The latter means that not all spins will satisfy the resonance condition ω_*s*_ = γ_*e*_*B*_tot_ = ω_K_. As a matter of fact, spins located at the position of the white dot in Figure 1e (where the coupling is maximum) will be far from resonance, therefore not contributing to the total signal. Independently of the magnitude of *B*_ap_, the resonance window for free paramagnetic spins will be fixed and it corresponds to the yellow volumes shown in Figure 1d.

Much more interesting for applications is the study of flux-closure topological magnetic textures, such as the magnetic vortex sketched in Figure 1g. Without requiring the application of external magnetic fields, vortex dynamics include the gyrotropic precession of the vortex core around its equilibrium position at frequencies in the range ω_v0_ ∼ 0.1–2 GHz. As an example, Figure 1h shows *B*_rms_ created by a R = 50 nm, *t* = 20 nm Py disc. We obtain very large *B*_rms_ close to 200 μT as in the case of the Py sphere of similar volume (see Figure 1e). Finally, we compute *B*_rms_ at 3 nm from the disc surface. Results obtained for discs of different sizes (*R* = 50 nm, 100 nm, and 400 nm and thicknesses given in the legend) and materials (CoFe, Py, and YIG) are shown in Figure 1i. The intensity of the zero point field fluctuations is comparable to that obtained with the saturated spheres, also following the expected *V*^–1/2^ dependence. Unlike the case described above, *B*_rms_ does not noticeably depend on *M*_sat_. In the following, we will analyze three distinct features that make vortex modes more suitable for spin detection compared to homogeneous modes.

#### (i) Absence of Biasing Field: The Resonance Window

Vortex modes do resonate in the absence of any external biasing field *B*_ap_ = 0, which is a great advantage compared with saturated ferromagnets. Besides, the demagnetizing stray field created by the vortex core itself reaches relatively large values close to the disc’s surface (see dark arrows in Figure 1g). This stray field is enough to make the vortex gyrotropic mode resonant with paramagnetic *S* = 1/2 spin fitting within the resonance window. In the case of the vortex, the latter takes the shape of a hollow semisphere (highlighted in yellow in Figure 1g). The total effective coupling *G* will be enhanced by a factor √*N* where *N* are the number of spins within the resonance window (see Implementation of the Vortex Sensor section).

**Figure 2:**
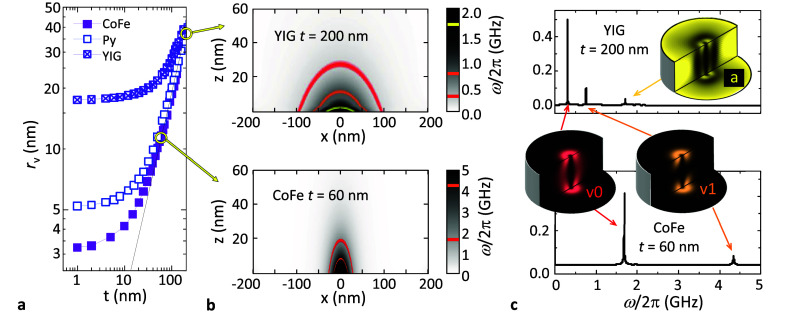
(a) Radius of the vortex core *r*_v_ vs *t* for *R* = 200 nm discs of different materials. *r*_v_ is nearly independent of *R*. (b) Spatial distribution of the resonance frequency for free *S* = 1/2 spins for YIG (top) and CoFe (bottom) discs with dimensions indicated. The latter is calculated as ω_*s*_ = γ_*e*_*B*_demag_. (c) Frequency spectrum of the same YIG (top) and CoFe (bottom) discs. The insets are the spatial FFT of the out-of-plane time-dependent magnetization of the different modes.

The size of the resonance window depends on the radius of the vortex core itself *r*_v_. The latter is given by the material-dependent exchange length  with *A* the exchange stiffness and μ_0_ the vacuum magnetic permeability. *r*_v_ is nearly constant, independent of the disc radius or thickness. However, for *t* ≫λ_ex_, *r*_v_ increases linearly with the thickness (see Figure 2a). As a result, the resonance window increases considerably for materials with low *M*_sat_ and a large thickness. This can be seen in Figure 2b, where we plot the spatial dependence of the spin resonance frequency ω_*s*_ = γ_*e*_*B*_tot_ for a paramagentic *S* = 1/2 spin as a function of its position above a *R* = 200 nm, *t* = 200 nm YIG disc and a *R* = 200 nm, *t* = 60 nm CoFe disc. The resonance window corresponding to the fundamental gyrotropic mode of the two discs is highlighted in red, i.e., the region for which ω_*s*_ = ω_v0_. As can be seen, the resonance window of YIG is considerably larger (diameter ∼190 nm and height ∼28 nm) than that of CoFe (∼54 nm × 19 nm). This stems from the (more than 1 order of magnitude) smaller *M*_sat_ of YIG compared to CoFe and the larger thickness of the YIG disc.

#### (ii) Independence on *M*_sat_ and Higher Order Modes

The vortex core gyro, key to produce the *B*_rms_, also yields flexure oscillations of the vortex core line across the disc thickness.^61,62^ For sufficiently thick discs, the latter leads to the emergence of higher order modes. This is shown in Figure 2c where we plot the frequency spectrum of the same discs shown in panel (b). Apart from the gyrotropic fundamental mode (denoted v0), we find a first flexure mode (v1). At higher energies, it is also easy to find azimuthal modes (a) that correspond to the rotation of two halves of the disc with opposite out-of-plane net magnetization. These modes can be distinguished in the FFT of the spatially resolved time-varying out-of-plane magnetization in the upper and bottom surfaces and the transverse cut of the discs, as shown in the inset of Figure 2c. Colored regions correspond to time-varying out-of-plane magnetization, whereas darker areas correspond to constant magnetization. The signatures of the vortex core precession can be seen in all modes.

Materials with low saturation magnetization are very interesting for sensing applications, as the lowest dampings are usually obtained in insulating magnets like YIG or V[TCNE]_*x*_. In this regard, the vortex mode offers an additional advantage over homogeneous Kittel modes for which . As shown in Figure 1i, spin coupling to vortex modes is nearly independent of *M*_sat_ encouraging the use of ultra low-damping ferrimagnets. However, low *M*_sat_ yields vortex gyration at sub-GHz frequencies, which is usually too low for practical readout circuits. In this way, flexure or azimuthal modes shall allow operating ultralow damping ferrimagnets at reasonable frequencies. As an example, see the upper panel in Figure 2b where we show the resonance windows (orange and yellow) for high frequency modes in a *R* = 200 nm, *t* = 200 nm YIG disc (ω_v1_ /2π = 760 MHz and ω_a_/2π = 1.7 GHz, respectively). Interestingly, the resulting spin–magnon coupling to high frequency modes is comparable to that of the fundamental mode. To illustrate this, we calculate the coupling for a single *S* = 1/2 spin located at the center, 3 nm above the surface of the YIG disc. This yields *g*_v0_/2π = 100 kHz, *g*_v1_/2π = 99 kHz, and *g*_va_/2π = 84 kHz for the fundamental, first flexure, and first azimuthal modes, respectively.

Additionally, high frequency modes in high *M*_sat_ materials would resonate with paramagnetic spins lying much closer to the disc surface, where the coupling is larger. This reduces the total volume of the resonance window, boosting the spatial resolution of the vortex sensing probe. For example, in the case of the *R* = 200 nm, *t* = 60 nm CoFe disc, the resonance window reduces from ∼54 × 19 nm (mode v0) down to ∼31 × 9 nm (mode v1).

#### (iii) Vortex Mobility: Scanning Spin Detector

**Figure 3:**
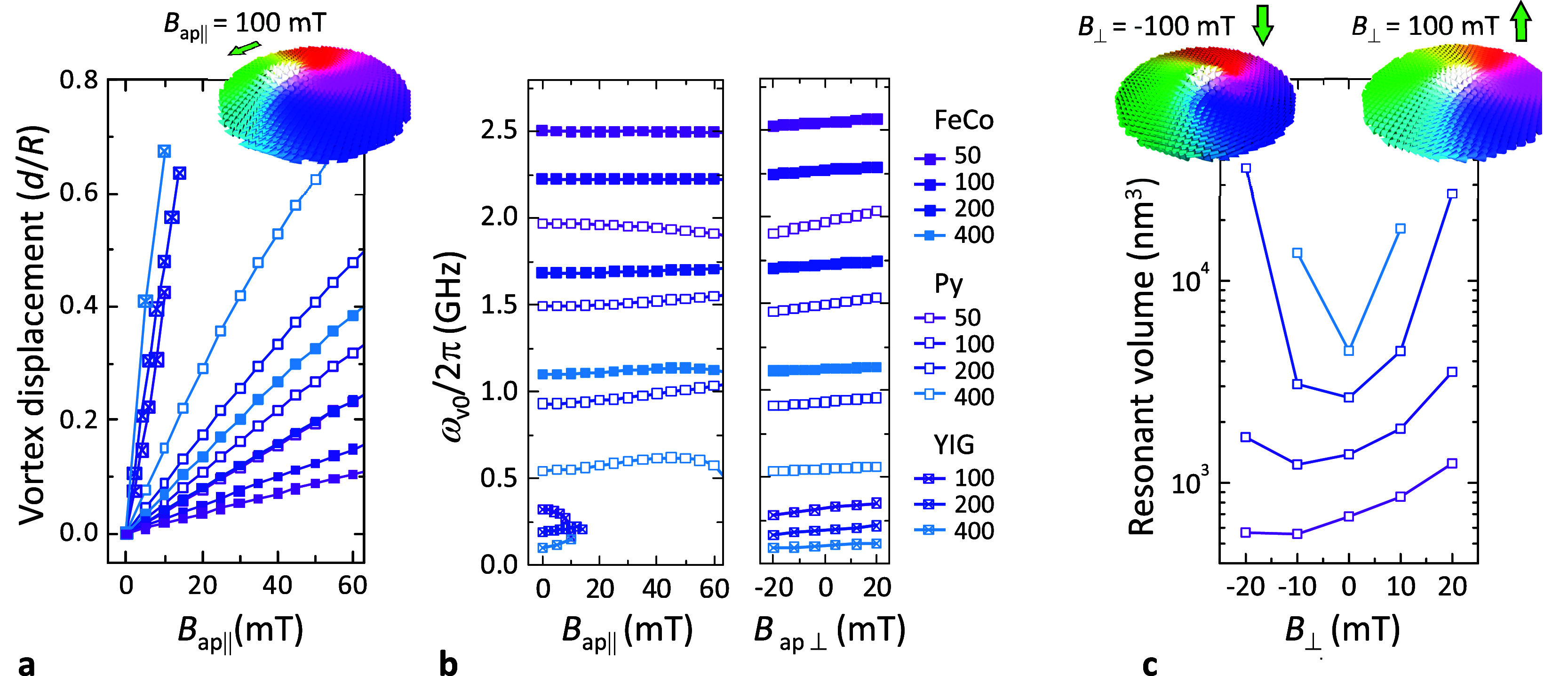
(a) Normalized displacement of the vortex core *d* upon an external in-plane bias field *B*_app∥_ and (inset) spatial distribution of the magnetization of a *R* = 50 nm Py disc under *B*_app∥_ = 100 mT. (b) Resonance frequency for the v0 mode vs the external in-plane *B*_app∥_ (left) and out-of-plane field *B*_app⊥_ (right). In all panels, simulations are made for *t* = 60 nm and different radius (in nm) and materials as indicated in the legend.

One of the most interesting properties of a spin sensor is the ability to scan over the surface of the sample. This is not trivial in the case of saturated ferromagnets but can be easily achieved in the case of magnetic vortices by using in-plane magnetic fields. The effect of an in-plane external field *B*_app∥_ is to enlarge the disc region having magnetization pointing in the same direction while decreasing the size of the region having opposite magnetization (see inset in Figure 3a). This results in an effective translation of the vortex core position *d* perpendicular to the direction of the *B*_app∥_. Figure 3a shows the numerically calculated values of *d* vs *B*_app∥_ for 60 nm-thick discs of different materials and radius. The vortex core moves progressively as *B*_app∥_ increases, until it approaches the edge of the disc where it is annihilated. We highlight that the vortex state remains stable up to the annihilation field. Experimental measurements indeed demonstrate that the energy barrier for vortex annihilation remain substantial, reaching several hundred K, even at large magnetic fields of several tens of mT.^54^ The latter holds true not only for discs of large radius but also for very small size, even below *R* = 100 nm. Additionally, the displacement of the vortex has little effect on the gyrotropic frequency as shown in Figure 3b (left).^63^ The coupling strength and the size of the resonance window does not vary notably as long as the vortex displacement *d* < *R*/2. For larger displacements, the resonance window is considerably enlarged so that vortex fluctuations still allow spin detection. The resonance can be also modified by means of an out-of-plane magnetic field *B*_app⊥_ as shown in Figure 3b (right).^64^ Interestingly, *B*_app⊥_ has also the effect of increasing (decreasing) the total size of the vortex core when applied parallel (antiparallel) to the vortex polarity. Combining these two effects, it is possible to tune the total size of the resonance window.

### Implementation of the Vortex Sensor

**Figure 4:**
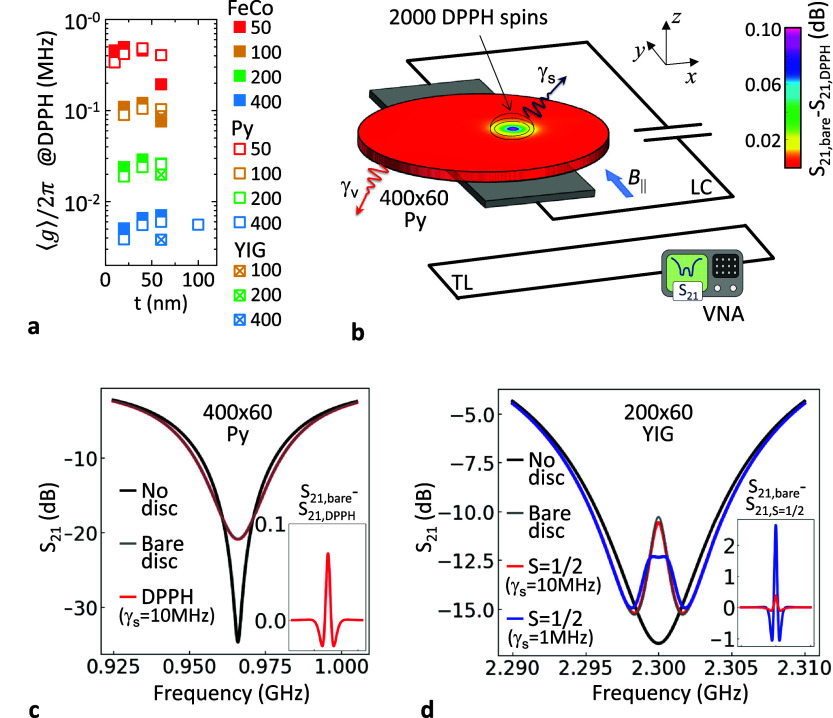
(a) ⟨*g*⟩ vs *t* for different materials and radius (nm) indicated in the legend. (b) A Py disc is located over a superconducting LC resonator coupled to a transmission line (TL) and the *S*_21_ parameter is measured. The density plot shows the difference between the transmission at resonance with and without a small drop of DPPH at *d* = 52 nm from the disc center. (c) Calculated *S*_21_ with no disc, with a bare Py disc and for a disc containing a drop of ∼2000 DPPH spins. The inset shows *S*_21,bare_ – *S*_21,DPPH_ in dB with the horizontal axis and scale being the same as in the main panel. (d) Transmission for a YIG disc (under identical conditions as in panel (a) with *B*_app⊥_ = 5 mT. *S*_21_ is calculated with no disc, with a bare YIG disc and with a disc containing a single spin *S* = 1/2 at 3 nm over the surface with line width γ_*s*_ = 10 MHz and 1 Mz. The inset shows *S*_21,bare_ – *S*_21,S=1/2_ in dB with the horizontal axis and scale being the same as in the main panel.

To finish, we discuss the experimental use of magnetic vortices to perform the nanoscopic scanning imaging of a spin ensemble. The total spin–magnon coupling can be calculated as , where *j* goes from 1 to *N* spins satisfying the resonance condition. The sensitivity relies on the ratio between *G* and both the losses on the vortex resonator (γ_v_) and the line width of paramagnetic spins (γ_*s*_). Increasing the coupling is achieved by using discs of small volume since *g* ∝ *V*^–1/2^ (cf. Figure 1i). However, the smaller the disc, the more difficult its fabrication, manipulation, and readout will be, so we will keep *R* ∼ 100–200 nm. The average coupling per spin defined as  does not depend noticeably on the disc thickness (see Figure 4a). For this reason, we will keep a relatively large *t* ∼ 60 nm, favoring the stabilization of vortices with not too small resonance frequencies.

If the total effective spin–magnon coupling satisfies *G* > γ_v_, γ_*s*_, we are in the strong coupling regime and the resonance peak of the vortex mode splits into two peaks separated by 2*G*. Measuring the splitting provides, therefore, a direct way of detecting the presence of spins as, e.g., the vortex core is scanned. On the other hand, sensing will be also possible in the weak coupling regime. In this case, the width of the vortex resonance γ_v_ will be enlarged if enough paramagnetic spins on the surface of the nanodisc satisfy the resonance condition. The line width γ_v_ can be experimentally measured using superconducting microcircuits that can be optimally coupled to magnonic resonators.^57,65−72^ In this way, the target spins can be deposited in powder or crystal form or from solution on the surface of the disc for nanoscopic EPR imaging.

A suitable experimental approach is depicted in Figure 4b. We consider a *R* = 200 nm, *t* = 60 nm Py disc that resonates at ω_v0_/2π = 0.93 GHz with losses γ_v_ = 14 MHz. We chose Py, although being a lossy ferromagnet, as it is probably the most common and easy material to fabricate and manipulate. The disc is located on the inductive part of a superconducting LC resonator of width 100 nm and thickness 50 nm, in good contact to it. The LC resonator is interrogated through a superconducting transmission line. Under such circumstances, the absorption dip produced by the resonant disc shall produce changes in the transmission that amount to several tens dB (see Figure 4c). Now, we take the case of a typical EPR calibrating sample of free-radical molecules of 2-diphenyl-1-picrylhydrazyl (DPPH) having ρ ∼ 2 spins/nm^2^, *S* = 1/2, *g*_*e*_ = 2, and γ_*s*_/2π ∼ 10 MHz. Scanning the vortex sensor, one could detect the presence of small drops containing 2 × 10^3^ DPPH spins (or, put in other words, volumes of only 0.4 attoliter with 2 spins per nm^2^), yielding *G*/2π = 1.2 MHz. The signature of coupling would be a variation of 0.1 dB of the resonance absorption as an external in-plane magnetic field is scanned (see Figure 4b and the inset in panel (c)). Even more interesting for sensing applications, the use of low-damping materials like YIG would allow the detection of single spins. For instance, the first flexure mode in a *R* = 100 nm *t* = 60 nm YIG disc would bring one single spin (lying at 3 nm over the surface) into resonance at ω_v1_/2π = 2.3 GHz, yielding *G*/2π = 0.7 MHz. For this purpose, an out-of-plane biasing field of 5 mT is necessary to tune the size of the resonance window. Under identical conditions as shown in Figure 4b and thanks to the low losses of YIG (γ_v_ = 0.6 MHz), this configuration would yield a large transmission signal, depending on the relaxation time of the spin. This is exemplified in Figure 4d where the transmission coefficient *S*_21_ resulting for a bare disc and a disc coupled to one individual spin with γ_*s*_/2π = 10 MHz (0.3 dB difference) and 1 MHz (3 dB difference) are compared.

## Conclusions

Our simulations suggest that, using Py, it is possible to detect small drops of 0.3 attoliter containing 2 spins per nm^2^ over a surface of 2π × 200^2^ nm^2^. Additionally, the vortex core can be easily scanned by means of external magnetic fields to perform EPR scanning microscopy. Interestingly, the same can be achieved in principle using dissipation-free spin currents. In this way, vortex-based EPR microscopy could be implemented *without* any externally applied magnetic field. High spin sensitivity stems from the small mode volume of the gyrotropic resonances which is independent of material parameters such as the saturation magnetization (cf. Figure 1i). This is an important advantage of the vortex probe sensor compared to saturated ferromagnetic nano-objects, encouraging the use of ultralow damping ferrimagnets that usually come with reduced saturation magnetization. This property, together with the possibility of using high frequency vortex modes, makes it possible to reach very large spin–magnon couplings. For example, a *R* = 100 nm YIG disc would allow detecting single spins with typical relaxation times of γ_*s*_ ∼ 10 MHz.

Our approach is also potentially very interesting to increase the weak interaction between superconducting microcircuits and spin qubits, e.g., molecular qudits based on single rare earth ions that offer transitions between tunnel-split ground state doublets with high spin.^73^ Using low-damping magnetic vortices could make it possible to read the state of individual spin qudits.^60^ Finally, we have also shown how to numerically normalize any magnon mode in ferromagnets of arbitrary size and shape. This can be used to calculate the zero-point magnetization fluctuations in confined nanomagnets including homogeneous magnon modes but also spin textures like domain walls, vortices, or skyrmions.

## Methods

### Micromagnetic Simulations

We use the finite difference software MUMAX3^74^ to solve the time-dependent Laudau–Lifshitz–Gilbert equation for a given sample geometry and material. The relevant material parameters are the saturation magnetization *M*_sat_, the exchange stiffness *A*, and the Gilbert damping α (CoFe: *M*_sat_ = 1.9 × 10^6^ A/m, *A* = 2.6 × 10^–11^ J/m, and α = 10^–3^; Py: *M*_sat_ = 0.86 × 10^6^ A/m, *A* = 1.3 × 10^–11^ J/m, and α = 1 × 10^–2^; YIG: *M*_sat_ = 0.14 × 10^6^ A/m, *A* = 0.37 × 10^–11^ J/m, and α = 10^–5^). In general, a disc of radius *R* and thickness *t* (or sphere of radius *R*) is simulated within a box with section 2*R* × 2*R* and thickness large enough to calculate the relevant stray fields at 100 nm above the surface of the ferromagnet. The lateral size of the cells (with volume *v*_cell_) is kept below o approximately equal to .

Magnon dynamics are simulated using a dc biasing field **B**_ap_ applied along a given direction, e.g., *x̂* in Figure 1d. *B*_ap_ is essential to obtain ferromagnetic resonances in the case of the saturated sphere but is unnecessary when dealing with vortices that also resonate at *B*_ap_ = 0. A sinusoidal time-dependent perturbation field **B** = β sinc(ω_cutoff_*t*) is applied perpendicularly to **B**_ap_ in the case of the sphere (*ŷ* direction) and parallel to the disc plane (*x*, *y*) in the case of vortices. This is equivalent to exciting all spin-waves at frequencies below ω_cutoff_. We identify the magnon modes by calculating the numerical FFT of the resulting time-dependent spatially averaged magnetization (see, e.g., Figure 2c). We can write the dynamics at each cell for every mode, either a Kittel (K) or a vortex (v) one, as
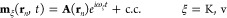
1with **r**_*n*_ the cell position, **A** (**r**_*n*_) the amplitude, and ω_ξ_ their frequency.

To calculate the spin–magnon coupling, we simulate the stray field, **B**_rms_(**r**_*j*_), generated by the (zero-point-fluctuations of the) vortex magnetization from which the coupling can be obtained:
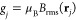
2For this purpose, we calculate the magnetic response on resonance using a perturbation field *B* = β sin(ω_ξ_*t*). Here ω_ξ_ is the mode frequency and β must be low enough to keep the system in the linear response regime, i.e., β < 200–500 nT. By doing so, we obtain, on the one side, the time-dependent vector magnetization at each cell *n* inside the ferromagnet. The amplitudes of **A** in eq 1) depend on the perturbation strength β. Therefore, we must *normalize* them to have single-magnon amplitudes. Using the usual formalism for magnon quantization the magnetization vector is normalized as^75^
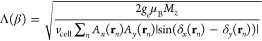
3Here, *M*_*z*_ is the spatial-averaged component of the magnetization along *B*_ap_ in the case of the sphere and out-of-plane in the case of vortices. *A*_α_(**r**_*n*_) are the perpendicular (the in-plane for the vortex case) amplitudes in eq 1 while δ_*x*_(**r**_*n*_) – δ_*y*_(**r**_*n*_) is the phase difference between these two components. On the other side, we obtain the stray magnetic field resulting at every spin position **r**_*j*_ outside the ferromagnet **B**_stray_(**r**_*j*_) = **B**_stray_^dc^(**r**_*j*_) + **B**_stray_^ac^(β, *t*; **r**_*j*_) where we have split the static and the time-dependent components and the dependencies on position, time, and perturbation field β are highlighted. The total zero-point field fluctuations are given by **B**_rms_(**r**_*j*_) = Λ**B**_stray_^ac^(**r**_*j*_), which is independent of β. Finally, we can calculate the contribution of **B**_rms_ able to induce spin transitions. The latter is given by  where *B*_rms,1_ and *B*_rms,2_ are the components of **B**_rms_ perpendicular to **B**_tot_(**r**_*j*_) = **B**_stray_^dc^(**r**_*j*_) + **B**_ap_ (see Results section). Inserting *B*_rms_ (**r**_*j*_) into (2), position-dependent spin-photon coupling can be obtained. We emphasize that Figure 1f,i shows the spatial dependence of *B*_rms_(**r**_*j*_), i.e., the part responsible for inducing spin transitions only.

The resonance window is also calculated numerically. For this purpose we consider only those spins for which the energetic criterion is satisfied, i.e., ω_*s*_ = γ_*e*_*B*_tot_ = ω_ξ_. This resonance window is enlarged by broadening of both the magnonic resonance and the spins. In general, it will be reasonable to consider those spins that satisfy:
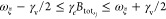
4with γ = max(γ_v_, γ_*s*_).

Finally, the average magnon–spin coupling per spin can be calculated as [cf. eq 2]:
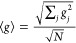
5where the sum considers only those cells within the resonance window. This is to say eq 5 is the sum of the position dependent coupling divided by the square root of the total number of spins that satisfy the resonance condition.

### Electromagnetic Simulations

In the case of the superconducting resonators, the spatial distribution of **B**_rms,*s*_ is calculated using the finite-element code 3D-MLSI.^76^ This software solves London equations in a superconducting circuit with given dimensions and a London penetration depth λ_L_. In the simulations, we set λ_L_ = 90 nm for Nb and a flowing current *i*_rms_. From here, 3D-MLSI allows calculation of the spatial distribution of supercurrents and the resulting **B**_rms,*s*_(**r**_*j*_). Under the particular geometry described in Figure 1a, all spins satisfy the resonance condition and *B*_rms,s_(**r**_*j*_) = |**B**_rms,s_(**r**_*j*_)|. The spatially dependent coupling is given by eq 2.

## Supporting Information Available

Theory for the vortex-sensor implementation: coupling between spins, vortex, LC-resonator and transmission line (PDF)
