# Supplementary material for: Scanning Spin Probe Based on Magnonic Vortex Quantum Cavities
Source: ACS Nano. 2024 Jan 25;18(6):4717–25. doi: 10.1021/acsnano.3c06704 (PMC10867890; doi:10.1021/acsnano.3c06704)
Supplement: Supplementary file 1 — nn3c06704_si_002.pdf [file nn3c06704_si_002.pdf]

# Supporting information for: scanning spin probe based on magnonic vortex quantum cavities

Carlos A. González-Gutiérrez,[†,‡,¶] David García-Pons,[†] David Zueco,[*,†] and María José Martínez-Pérez[*,†]

†*Instituto de Nanociencia y Materiales de Aragón (INMA), CSIC-Universidad de Zaragoza, Zaragoza, ES-50009 Spain*

‡*Department of Physics and Applied Physics, University of Massachusetts, Lowell, MA 01854 USA*

¶*Instituto de Ciencias Físicas, Universidad Nacional Autónoma de México, Av. Universidad s/n, Cuernavaca, Morelos, 62210, México*

E-mail: dzueco@unizar.es; pemar@unizar.es

## Theory for the vortex-sensor implementation: coupling between spins, vortex, LC-resonator and transmission line.

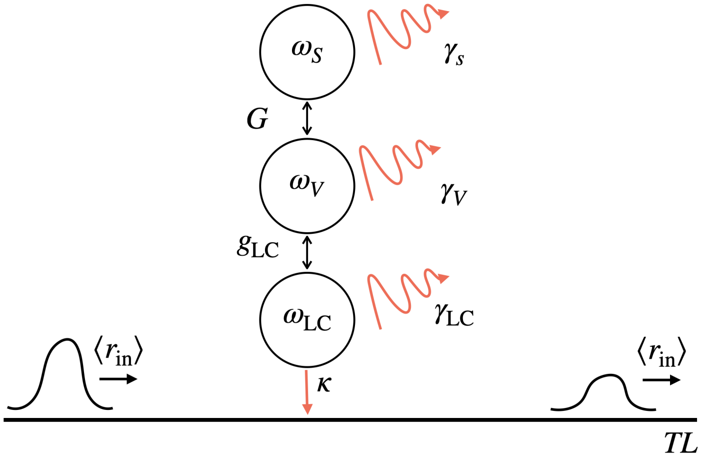


Figure S 1: Sketch of a transmission experiment. The input signal is sent through a transmission line interacting with the LC-circuit. In this sketch the modes (the LC-resonator, the vortex and the spins) are represented as circles, the couplings and dissipation channels are indicated. The dynamics of the model is given in Eq. (3).

The experimental setup for spin detection discussed in the main text involves a LC resonator coupled to a transmission line. Simultaneously, the disc is coupled to the LC resonator (mounted on top of the inductor), as shown in Fig. 4 in the main text. The stabilized vortex scans the spins. The spin detection relies on the coupling of the latter to the vortex, resolved through a transmission experiment, that we model here.

An input coherent signal is sent through the transmission line, and the output is measured after interaction with the complete system: LC resonator + disc + spins. For convenience and clarity, we illustrate the coupling topology in Fig. S1. We observe that the LC is directly coupled to the TL. Thus, employing input-output theory, this transmission is given by:

$$S_{21}(\omega) = \frac{\langle r_{\text{out}} \rangle}{\langle r_{\text{in}} \rangle} = 1 - \kappa \chi_a(\omega) \, . \tag{1}$$

Here, $\langle r_{\text{in,out}} \rangle$ represents the averages of the input and output right-moving fields, $\kappa$ quantifies the

coupling to the TL, and $\chi_a$ is the LC response, which can be computed as:

$$\chi_a(\omega) = \frac{\langle a \rangle}{\sqrt{\kappa}\alpha_{\text{in}}} \,. \tag{2}$$

Here, $\langle r_{\text{in}} \rangle = \alpha_{\text{in}} e^{-i\omega t}$. Hence, according to the input-output theory, the transmission reduces to the calculation of the LC response function in this case. To compute the LC response function, we employ a coupled mode model, depicted in Fig. S1, where the LC, vortex, and spin are modeled as harmonic modes coupled among themselves. The dynamics are given by:

$$\frac{d}{dt}\begin{pmatrix} \langle a \rangle \\ \langle a_V \rangle \\ \langle a_S \rangle \end{pmatrix} = \begin{pmatrix} -i\omega_{\text{LC}} - \kappa - \gamma_{\text{LC}} & -ig_{\text{LC}} & 0 \\ -ig_{\text{LC}} & -i\omega_V - \gamma_{\text{v}} & -iG \\ 0 & -iG & -i\omega_S - \gamma_S \end{pmatrix} \begin{pmatrix} \langle a \rangle \\ \langle a_V \rangle \\ \langle a_S \rangle \end{pmatrix} + \begin{pmatrix} -i\sqrt{\kappa}\alpha_{\text{in}} e^{-i\omega t} \\ 0 \\ 0 \end{pmatrix} \tag{3}$$

Here, $\langle a \rangle$, $\langle a_V \rangle$, and $\langle a_S \rangle$ represent the modes for the LC resonator, vortex, and spins, respectively. The remaining parameters include the LC-vortex coupling, $g_{\text{LC}}$, the (collective) spin-vortex coupling, $G$, and the line-LC coupling, $\kappa$. Finally, $\gamma_{\text{LC}}$ denotes the intrinsic losses of the resonator. The vortex and spin dissipation, $\gamma_{\text{v}}$ and $\gamma_s$, are also taken into account in the model. Equation (3) represents a linear set of differential equations that can be solved by moving to the interaction picture $\langle a \rangle \to \langle a \rangle e^{-i\omega t}$. This transformation yields an algebraic set of three coupled linear equations, which admit an analytical solution from which $\langle a \rangle$ can be obtained. Consequently, $S_{21}$ in Eqs. (1) and (2) is given by:

$$S_{21}(\omega) = 1 - \frac{\kappa}{\kappa + \gamma_{\text{LC}} + i(\omega_{\text{LC}} - \omega) + g_{\text{LC}}^2 \frac{\gamma_S + i(\omega_S - \omega)}{G^2 + \left(\gamma_S + i(\omega_S - \omega)\right)\left(\gamma_{\text{v}} + i(\omega_V - \omega)\right)}} \,. \tag{4}$$

The typical limiting cases of $G = g_{\text{LC}} = 0$ (just the resonator) or the resonator plus vortex $G = 0$ are easily recognized.

The resonance frequency of the superconducting cavity is given by $\omega_{\text{LC}} = 1/\sqrt{LC}$ with $L$ and $C$ the inductive and capacitive components of the circuit, respectively. $L$ can be tuned by flux-biasing

a superconducting quantum interference device (SQUID) coupled in series to the inductive part of the resonator.[1,2] In this way, $\omega_{\rm LC}$ can be adjusted so that $\omega_{\rm LC} = \omega_{\rm v0}$. The latter is true even if a dc magnetic field is applied along the disc plane. Under these circumstances, the vortex resonance frequency $\omega_{\rm v0}$ varies slightly (a few 10 MHz at maximum) as shown in Fig. 3 in the main text. On the other side, $\kappa$ is fixed by design and typically takes values within a few kHz up to $\sim$ 100 MHz. The value of $\kappa$ will determine the maximum quality factor of the resonator $Q = \omega_{\rm LC}/\gamma_{\rm LC}$. In our simulations we use $\kappa/2\pi = 30$ MHz and $Q = 10^3$ which is feasible.

The magnetic disc behaves as a resonator with characteristic frequency $\omega_{\rm v0}$ set by the aspect ratio and saturation magnetization $M_{\rm sat}$. Its loses are mainly given by the Gilbert damping $\alpha$ of the ferromagnetic material but they also depend on the particular excited mode. We determine both $\omega_{\rm v0}$ and $\gamma_{\rm v}$ by means of micromagnetic simulations with MUMAX3. Here, we use $M_{\rm sat}$, $\alpha$ and exchange stiffness $A$ according to literature values for each material, as summarized in the **Methods** Section.

The coupling between the vortex and the superconducting resonator is calculated as described in Ref.[3,4] and briefly summarized here. We first calculate the zero point current fluctuations flowing though the LC resonator:

$$i_{\rm rms} = \omega_{\rm p}\sqrt{\frac{\hbar\pi}{4Z_0}}, \tag{5}$$

with $Z_0$ the impedance. Next, we estimate the modulus of the zero point field fluctuations $B_{\rm rms,s}$ produced by $i_{\rm rms}$ at the vortex center position. $B_{\rm rms,s}$ depends on the thickness and width of the superconducting line. Finally, the coupling is calculated as

$$g_{\rm LC}^2 = \frac{\kappa B_{\rm rms,s}\Delta M V}{4\hbar} \tag{6}$$

where $V$ is the volume of the magnetic disc and $\Delta M$ is the amplitude of the volume averaged magnetization modulation when the vortex is excited with a varying magnetic field of amplitude $b_{\rm rms,s}$ at frequency $\omega_{\rm v0}$. The validity of this equation has been demonstrated by comparing nu-

merical simulations based on (6) with experimental coupling values from transmission and cavity measurements with Py nanomagnets.[5]

In our simulations we use 3D-MLSI to estimate the distribution of supercurrents in the superconducting circuits and the resulting magnetic fields. Here, we have assumed superconducting Nb circuits with thickness 50 nm and width 100 nm. The impedance of the LC resonator can be set by design and is assumed to be $Z_0 = 10\ \Omega$. By doing so, in the particular cases described in the main text we obtain $g_{\rm LC}/2\pi = 6.8$ MHz for the 400 nm $\times$60 nm Py disc and $g_{\rm LC}/2\pi = 1.4$ MHz for the 200 nm $\times$60 nm YIG disc.

Finally, we assume that each spin is resonant with the vortex at frequency $\omega_{\rm v0} = \omega_S = \gamma_e B_{\rm tot}$ with $B_{\rm tot} = B_{\rm ap} + B_{\rm stray}$ the total static field at each spin position. $G$ is calculated as described in the main text.

# References


(1) Planat, L.; Al-Tavil, E.; Martínez, J. P.; Dassonneville, R.; Foroughi, F.; Léger, S.; Bharadwaj, K.; Delaforce, J.; Milchakov, V.; Naud, C.; Buisson, O.; Hasch-Guichard, W.; Roch, N. Fabrication and Characterization of Aluminum SQUID Transmission Lines. *Physical Review Applied* **2019**, *12*, 064017.

(2) Uhl, K.; Hackenbeck, D.; Füger, C.; Kleiner, R.; Koelle, D.; Bothner, D. A flux-tunable YBa2Cu3O7 quantum interference microwave circuit. *Applied Physics Letters* **2023**, *122*.

(3) Martínez-Pérez, M. J.; Zueco, D. Strong Coupling of a Single Photon to a Magnetic Vortex. *ACS Photonics* **2018**, *6*, 360–367.

(4) Martínez-Pérez, M. J.; Zueco, D. Quantum electrodynamics with magnetic textures. *New Journal of Physics* **2019**, *21*, 115002.

(5) del Rincón, S. M.-L.; Gimeno, I.; Pérez-Bailón, J.; Rollano, V.; Luis, F.; Zueco, D.; Martínez-

Pérez, M. J. Measuring the Magnon-Photon Coupling in Shaped Ferromagnets: Tuning of the Resonance Frequency. *Physical Review Applied* **2023**, *19*, 014002.
